# Notes and Comments

**Published:** 1921-11

**Authors:** 


THE
### HOSPITAL and HEALTH REVIEW

### NOTES AND COMMENTS.

From the recent increase in the number of notifications of scarlet fever it seems possible that we are likely to suffer from another outbreak of this disease during the autumn and winter, as large perhaps as that which occurred a year ago. Scarlet fever to-day is a mild disease and causes very few deaths. Compared with measles, whooping-cough, and summer diarrhœa it is relatively a trivial complaint. We see no longer those fatal septic cases, which were common twenty and more years ago; indeed, to-day the disease is so mild as to be, at times, most difficult of diagnosis. It is only when desquamation occurs that the diagnosis can accurately be made in a large number of instances. Scarlet fever is one of those diseases of which the type appears to alter—during one period the mortality is low, and at other times the infection is most virulent, with a mortality as high as 30 per cent. Dublin in the years 1801-1802 suffered from a severe and fatal epidemic; then followed on this many years when the disease was prevalent, but the mortality was trifling; and then in 1834-1835 the type of the disease altered and became again very virulent. In this country to-day we are passing through a mild phase of the disease; indeed, the increased prevalence of scarlet fever is an evidence of this—the disease is so inconspicuous that there are many "missed cases" that spread the infection. With so many undiagnosed cases not isolated, it is not surprising that the isolation of the remainder has not served to check the incidence of the disease.

Very properly local authorities made provision for the isolation and treatment of scarlet fever when that disease was severe in its effects and easily diagnosed by parents and doctors. To-day it may be contended that isolation hospital treatment is unnecessary, except for the occasional severe case or for the mild case that develops complications, such as nephritis or mastoiditis. It would be sufficient if the mild cases were nursed at home, by a health visitor perhaps; and it is not likely that there would be any additional risk of the home-nursed disease becoming complicated, any more than there is for the "missed" case. We have heard the argument that the present non-severity of scarlet fever is due to the removal of cases to hospital; but this we doubt. The disease has its ups and downs of severity as it had in Dublin a hundred years or more ago when isolation hospitals had not been instituted.

The large numbers of beds liberated in isolation hospitals, if this policy of excluding scarlet fever were adopted, could be used for the treatment of diseases which are really serious. Not many local authorities are able to give much hospital accommodation to cases of summer diarrhœa, whooping-cough, and measles; and if we really wish to save life and to lessen the ravages of diseases that lead to so much subsequent enfeeblement, it is surely better to devote our hospital beds and our energies to these diseases rather than to one which so seldom cripples or kills. In the years 1861-1865 there was an average of 982 deaths in England and Wales per million persons living from scarlet fever; but in the years 1911-1920 scarlet fever caused only forty-seven deaths per million of the population on the average, whereas measles caused 270 deaths, whooping-cough 184 deaths, and epidemic diarrhœa a yearly average of 172 deaths per million. These figures give some idea of the relative importance of these diseases, and we must remember, in addition, that for every death from whooping-cough, measles, or diarrhœa there are many wounded children left alive. The reception of those more serious diseases, instead of scarlet fever, into our hospitals would render these illnesses less fatal and disastrous; and it would have another and excellent effect—more than in any other way it would bring home to the parents the seriousness of these "children's ailments," and

**NOTES AND COMMENTS**—(*cont.*).

in the popular imagination they would come at once to their true place among the important and severe diseases. Against the exclusion of cases of scarlet fever from hospital there is, however, one objection: if all cases were nursed at home the wage-earners of the family might not be able to go to work—indeed, they themselves might contract the disease, since adults are more frequently attacked by scarlet fever than they are by measles and whooping-cough. The whole question is one which would well bear discussion, since it is a matter of public health and economic interest.

---

The Oswestry Board of Guardians has had to consider the relation of the hospital to the "house," or, in other words, of the superintendent nurse to the staff of the workhouse master. Though the details are minor, they illustrate the perpetual disadvantage of the non-separated infirmary. The superintendent nurse wants the guardians to engage three outside charwomen that she may have no longer to rely on the inmates to assist in this work. The inmates, in effect, will not serve under two masters, their own chief and the superintendent nurse. Friction has resulted, and if her request be not granted the superintendent nurse offers to resign. Experience has abundantly proved that the only solution of such difficulties is the complete separation of the infirmary from the workhouse. It is the only solution here; whether or not the guardians postpone that solution it will have to come. The time has gone by for the continuance of the non-separated infirmary, especially when, as at Oswestry, the latter has as many as one hundred beds.

---

At the meeting of the Church Congress at Birmingham, last month, Lord Dawson of Penn spoke eloquently in favour of consideration being given to the various sociological and religious problems underlying the subject of birth-control. The practice of limiting conception has, in Lord Dawson's opinion, come to stay; it is an established fact, and for good or evil it has to be accepted. Like other innovations, this new economic and sociological factor has its heated opponents and its keen advocates. Those of our readers who are familiar with the work of the French dramatist Brieux will remember that he has written two plays, the one denouncing the evil of too large a family and the other showing the peril of a married couple having no children. The dramatist leaves his readers to infer that there is a happy mean between these two extremes, and in this we are in agreement both with him and Lord Dawson. The childless marriage and the tragedy of the marriage that results in only one child are familiar to us all; of the two the latter is sometimes the more terrible. On the other hand, we have the woman worn out with maternity, the quiver not only full but overflowing, and the children, not like olive branches, but as an untended and uncared-for forest round about the table. And with the excess of unwanted lives comes the inevitable mortality—"After I'd had ten of them I didn't care whether they died or not"! Those in favour of birth-control and those opposed to the ethics and practice of it will, no doubt, find weighty arguments that appear to give support to their cases, and there will, perhaps, be much ill-feeling and personal bitterness, at which the people as a whole will be mildly amused. No doubt we shall see, as we have seen on the subject of the control of venereal diseases, the unedifying spectacle of one section of the medical profession and of the religious bodies being wordily opposed by another section of the saints and doctors, and we shall hear great argument about it and about. Each camp will call upon science and Christianity in support of its case, and the net result will be this—that in the publicity given to all this wrangling and hair-splitting the public will learn quite a lot about the principles and practice of birth-control.

---

The appointment of financial advisers to hospitals, to help them in raising funds at this critical time, is a growing practice. Since the raising of money for charities is at least as much a personal art as a public business, very much depends upon the man. We therefore read with interest the first speech made by Mr. Isaac Edwards, who has been appointed financial adviser to the Merthyr General Hospital. He reminded his audience that he was virtually asked "to find the money" needed, but that the attitude of a financial adviser could not be so crudely expressed. He had a good deal of experience in raising money within and without the borough, and he had learnt from it that tact, sympathy, and the avoidance of "feeling" were essentials. To create a good spirit friends must be made, for only by making friends with those with money to give could they be induced to put their hands in their pockets. "It requires cleverness, tact, and ability to induce a man to open his pocket when the control of that pocket is in his own hands." He proposed, then, to make friends for the hospital, because a group of such friends would begin to create a civic interest and a communal pride in the institution. The result is to make those not on the list of subscribers feel that they have not done their duty. Class feeling, irritation, must be avoided; demands must not be made, but good feeling created. These words are simple, sincere, and the fruit of experience. That they are worth quoting is apparent, because, however simple they seem, very few people use them in this interesting and direct way.

---

It is difficult to dogmatise on such questions, but the number of street accidents seems to increase, at least in certain places, and the increase, whether local or general, calls attention to a matter of some importance. These accident cases do not all require out-patient treatment. A proportion, costly to the hospital, requires admission to the wards; and in respect of such cases very little support is forthcoming. In the old days accident cases were merely part of the day's work for a

**NOTES AND COMMENTS—**(*cont.*).

hospital. From a medical point of view they will always remain so. But legislation has altered the position of these cases in the economic sphere. In the old days few of these cases were insured. To-day insurance is the rule, and not only is it the rule, but the injured as often as not are covered by insurance for third-party risks. Consequently, the hospital is now of use not merely to the injured but to the third party which insures him and to the insurance company as well. Clearly, therefore, the hospitals are entitled to some recompense; and this should preferably be sought from the insurance companies. Since the report of isolated cases would probably fall on deaf ears, might not a useful beginning be made by forming a list of all accident cases so insured, and at the end of the year reporting to each company concerned the number of cases covered by it? In this way impressive evidence might be collected of the amount of unacknowledged service which each hospital performs for the different companies.

---

Some correspondence has recently appeared in "The Times" on the subject of the operative treatment of enlarged tonsils and adenoids. A retired Lieutenant-Colonel of the R.A.M.C. wrote in the first place to say that the operation was of no value, that actual harm might be done by operating, and that enlarged tonsils and adenoids were better left alone or treated by means other than operation. Of course, there were some replies to his remarks from surgeons and others who appreciate the benefit of this operation. From the correspondence we imagine that there must be two opinions about the value of operative treatment for these truly crippling conditions, but we believe that those who are in favour of inaction, of allowing the children slowly to become diseased and deaf, are but few in number. The Croix-Rouge Américaine have recently circulated in France some admirable picture postcards, one of which portrays an adenoid child. Under the picture are the words: "Votre bébé respire-t-il par la bouche? A-t-il des maux d'oreilles? Ronfle-t-il en dormant? . . . S'enrhume-t-il facilement? . . . Si oui; consultez le médecin car il peut être nécessaire de lui enlever les amygdales ou des végétations adénoïdes . . . L'opération est inoffensive." We would commend this postcard to the retired Lieutenant-Colonel.

---

As we anticipated, the formation of Local Committees under the Voluntary Hospitals Commission is not unattended with difficulties. From the reports which reach us it is clear that the perhaps unavoidable delay is irksome to those hospitals which are counting, perhaps hastily, upon a share of the Government grant. The delay by itself would not be unduly important were not the functions of the new Local Committees still often misunderstood. Mr. W. F. H. Thomson, when presiding over the quarterly meeting of the York County Hospital, lately combated the suggestion that "No hospital which tried to help itself would get anything." The very reverse is the truth. But that this notion should need correction is sufficient perhaps to show that the individual hospitals often are thinking too much of a share of the Government grant and too little of their duties of co-operation with other local hospitals, the fulfilment of which can best entitle them to it. As we have often said, hospitals still think too exclusively of their own immediate needs, and have yet to learn to think of their share in the work common to their area. For this reason the new Committees were not designed to represent the hospitals alone, but rather to gather together those best able to grasp the hospital problem in the local area.

---

The Local Committees which will make recommendations for grants will make them in the light of the willingness of each hospital to co-operate with its neighbours, and loyally to carry out the common policy which the Local Committee will advise. Its power of the purse is designed at least as much to secure respect for its recommendations as to benefit the particular hospitals. We therefore hope that the hospitals will look beyond the possible grant that they may receive to the object of the grant itself, which will be the participation of each local hospital in the common policy for all in each area. The Regional Committees of the British Hospitals Associations can do useful work at present in helping to educate the hospitals in this elementary attitude of mind. For under the new conditions a hospital can help itself best by co-operating with neighbouring institutions in an agreed campaign of work and a common financial policy. To this end the new Local Committees should provide the leadership and the ideas, loyalty to which will secure a recommendation for a grant far more readily than the presentation of a claim for money or the insistence on a "private" indebtedness to the bank. Once the Committees have been formed in each area the hospitals should look to them for advice and leadership, even more than they should look to them for funds.

---

The Council of the Metropolitan Borough of St. Pancras decided recently to dismiss from its service Dr. Gladys Miall-Smith, an assistant medical officer for maternity and child-welfare work. The Council had previously passed a resolution terminating the appointments of certain married women in its employ whose husbands were in receipt of salaries fully sufficient to support their wives; and it would seem that Dr. Miall-Smith came within this category, in the opinion of the Council, for she had been married since her appointment in 1920. At the first consideration a dismissal for an act that was certainly no offence might seem to be a real injustice, and Dr. Miall-Smith has submitted several arguments on her own behalf. No mention was made in the advertisement of the appointment that marriage was a disqualification, and, indeed, the selection committee interviewed, among the other applicants, a married woman; again, the work that Dr. Miall-Smith has performed since her appoint-

**NOTES AND COMMENTS**—*(cont.)*.

ment is acknowledged to have been excellent, and there was no reason to suppose that marriage would prejudice the admirable quality of her work. She pointed out to the committee that there is no unemployment problem at the present time among medical women, and that therefore the question of her keeping a single woman out of employment does not arise. These are weighty arguments; and there are others to support, not only this particular case, but the undesirability in general of dismissing women from appointments because they are married. On the other side it can be said, however, in this instance, that this lady doctor had no promise of security of tenure, and that the St. Pancras Council, having passed its general resolution regarding married women, is right in seeing that its wishes are obeyed. It is a hopeless business to begin making exceptions in matters of discipline. To those of our readers who have read "The Young Visiters" we suggest that the St. Pancras Borough Council is rather in the position of Mr. Salteena in the cab. "Sit down said Ethel you are standing on my luggage. Well I am paying for the cab said Mr. S. so I might be allowed to put my feet where I like."

---

The strong woman at the fair, who juggles with grand pianos and elephants, may make more money than many a brain-worker, and the fees paid to professional footballers and boxers are sometimes out of all proportion to the public utility of these gentlemen; but, on the whole, it can be said that these high wages paid for muscle are exceptional, and it is the general rule to pay a man according to his brain- rather than his horse-power. Samson, Hercules, and a few other strong men have survived in memory or tradition; but the world has always valued its thinkers more than those who are merely physically strong, and the names that have survived of the men of intellect are legion compared to the small number of the athletes that are remembered. The verdict of the centuries is in favour of the value of the thinker.

---

The intellectual man, whose brain is highly trained for his own line of special work, is paid well—firstly, for his expert knowledge, and, secondly, for the responsibility that he is called upon to bear. To be the medical superintendent of a hospital or to be a medical officer of health is laborious work; the working day of such a one is twenty-four hours long, in that he is never "off duty" except when he is on a holiday; and, worse than that, responsibility is shifted on to him by others, and he cannot transfer it to someone else. The reward of one in a position of responsibility is to see his work progressing smoothly and regularly, and to know that because of his greater capacity he will receive a reasonable salary.

---

Then mark what sometimes happens in a hospital or a health department. A professional man is engaged at a reasonably high salary to undertake certain work for which his brain is specially adapted or trained; but when he enters on his duties he has other work thrust upon him, to which he is unsuited, which robs him of time that is valuable to his employers. For example, the organising secretary of a hospital may have to address envelopes or lick stamps. A town clerk may of necessity be forced to do much petty secretarial work. A medical officer of health may have to carry out office routine that could well be done by a lad who has just left school. It is a great waste of public money to pay a man an expert's salary and then to expect him to occupy his time in doing the work of an office boy. We heard recently of an assistant medical officer of health who was told to go out into the country ten miles or so away and inspect a village. He was informed that he could walk there and back again, because there were no trains and car hire was expensive. As he came home weary at the end of his work, he probably considered that he had wasted his day—five or six hours of it at the least. We hear a lot of talk about economy; but we shall see less real waste when our brain-workers are allowed to do the work for which we pay them.

---

The appointment of an officer in charge of the hospital at Holloway Prison has been the subject of a question by Sir Samuel Hoare in the House of Commons, who has elicited from the Home Secretary that the officer selected has had "much nursing experience," has been "engaged on hospital duties for seventeen years," and is reported by the medical officers to be fully qualified and very capable. He further stated that the Nursing Board was not consulted as to the appointment. nor have they made any representation on the matter. It is difficult to credit that the Nursing Board, which was appointed by the Prison Commission early in the year, and includes such experienced matrons as Dame Sarah Swift, Miss Hogg of Guy's, Miss McIntosh of St. Bartholomew's, and Miss Jolley, the Hospital Lady Superintendent of Holloway Prison, should have made no representation on the subject. This is a matter which vitally concerns them, for it cuts at the root of the whole nursing administration and the care of the sick in our prisons.

---

Our readers will be interested to see the result of our leaflet competition, which we publish on page 58. We had no idea when we asked our readers to invent a leaflet on the care of the teeth that so much talent for that class of writing existed in the country. This month we are offering three guineas to the correspondent who sends in the best new and original leaflet on the subject of *Cleanliness*. This leaflet, like the last, must be suitable for circulation among school-children. Leaflets which begin with the statement that "Cleanliness is next to godliness" will be put into the wastepaper basket, for every child is sick of hearing that phrase, and would be discouraged by it from the further perusal of the leaflet. Details of this competition will be found on page 59.

